# Transgenic Mice Expressing MCP-1 by the Urothelium Demonstrate Bladder Hypersensitivity, Pelvic Pain and Voiding Dysfunction: A Multidisciplinary Approach to the Study of Chronic Pelvic Pain Research Network Animal Model Study

**DOI:** 10.1371/journal.pone.0163829

**Published:** 2016-09-29

**Authors:** Suming Xu, Xu Wang, Yaoqin Wang, Susan Lutgendorf, Catherine Bradley, Andrew Schrepf, Karl Kreder, Michael O'Donnell, Yi Luo

**Affiliations:** 1 Department of Urology, University of Iowa, Iowa City, Iowa, United States of America; 2 Department of Psychology, University of Iowa, Iowa City, Iowa, United States of America; 3 Department of Obstetrics and Gynecology, University of Iowa, Iowa City, Iowa, United States of America; Cedars-Sinai Medical Center, UNITED STATES

## Abstract

Monocyte chemoattractant protein-1 (MCP-1) is one of the key chemokines that play important roles in diverse inflammatory and chronic pain conditions. Interstitial cystitis/bladder pain syndrome (IC/BPS) is a chronic and debilitating inflammatory condition of the urinary bladder characterized by the hallmark symptoms of pelvic pain and voiding dysfunction. To facilitate IC/BPS research, we used transgenic technology to develop a novel urothelial MCP-1 secretion mouse model (URO-MCP-1). A transgene consisting of the uroplakin II gene promoter and the mouse MCP-1 coding sequence with a secretory element was constructed and microinjected. URO-MCP-1 mice were found to express MCP-1 mRNA in the bladder epithelium and MCP-1 protein in the urine, and developed bladder inflammation 24 hours after intravesical administration of a single sub-noxious dose of lipopolysaccharide (LPS). The inflamed bladders of URO-MCP-1 mice exhibited elevated mRNAs for interleukin (IL)-1ß, IL-6, substance P precursor, and nerve growth factor as well as increased macrophage infiltration. In parallel with these phenotypic changes, URO-MCP-1 mice manifested significant functional changes at days 1 and 3 after cystitis induction. These functional changes included pelvic pain as measured by von Frey filament stimulation and voiding dysfunction (increased urinary frequency, reduced average volume voided per micturition, and reduced maximum volume voided per micturition) as measured by micturition cages. Micturition changes remained evident at day 7 after cystitis induction, although these changes were not statistically significant. Control wild-type C57BL/6 mice manifested no clear changes in histological, biochemical and behavioral features after similar cystitis induction with LPS. Taken together, our results indicate that URO-MCP-1 mice are hypersensitive to bladder irritants such as LPS and develop pelvic pain and voiding dysfunction upon cystitis induction, providing a novel model for IC/BPS research.

## Introduction

Aberrant overexpression of monocyte chemoattractant protein-1 (MCP-1; also named CCL2) has been observed in diverse inflammatory and chronic pain conditions [1]. MCP-1 is produced by multiple cell types including bladder epithelial cells [1,2] and plays an important role in recruiting monocytes/macrophages as well as other leukocytes to sites during an inflammatory process. The fundamental importance of MCP-1 and its cognate receptor CCR2 are underscored by studies using genetic knockout mice lacking either of these two proteins. These genetically deficient mice display decreased macrophage recruitment and activation, increased susceptibility to mucosal infection, and reduced T cell responses [3–5]. Moreover, transgenic models of tissue specific MCP-1 expression have recapitulated many inflammatory disorders such as insulitis, pneumonitis and encephalitis but typically only after an additional inflammatory stimulus is introduced [6–9].

In humans, elevated MCP-1 has been observed in various inflammation and autoimmune associated diseases such as inflammatory bowel disease, multiple sclerosis, diabetes, rheumatoid arthritis, and allergic asthma [1]. Elevated MCP-1 has also been reported for overactive bladder (OAB) [10,11] and chronic prostatitis/chronic pelvic pain syndrome (CP/CPPS) [12]. Similarly, animal studies have demonstrated elevated MCP-1 in bladder outlet and ureteral obstruction, which correlate with bladder and renal pathology, respectively [13,14]. In addition, elevated MCP-1 in the bladder has been observed to associate with bladder inflammation, reduced bladder capacity, and pelvic pain in a cyclophosphamide (CYP)-induced cystitis model [15]. Moreover, one study demonstrated that elevated MCP-1 in the bladder promoted histamine release from mast cells in a protamine sulfate and lipopolysaccharide (LPS)-induced cystitis model [16]. Another study demonstrated that mast cell associated MCP-1 mediated bladder inflammation and chronic pelvic pain in a uroplakin peptide-induced autoimmune cystitis model [17]. All these findings suggest that aberrant overexpression of MCP-1 may contribute to pelvic pain and voiding dysfunction in interstitial cystitis/bladder pain syndrome (IC/BPS), a chronic and debilitating inflammatory condition of the urinary bladder characterized by the hallmark symptoms of pelvic pain and voiding dysfunction [18]. Here we report the development of a transgenic mouse model (URO-MCP-1) that secretes MCP-1 by the bladder epithelium and develops bladder inflammation, pelvic pain and voiding dysfunction upon intravesical administration of a single sub-noxious dose of LPS. The URO-MCP-1 model demonstrates the hallmark symptoms of IC/BPS and provides a novel model for IC/BPS research.

## Materials and Methods

### Ethics statement

All animal experiments were approved by University of Iowa Animal Care and Use Committee (Permit Number: 1308153) and performed according to the Guide for the Care and Use of Laboratory Animals of the National Institutes of Health.

### Generation of URO-MCP-1 transgenic mice

**Fig 1:**
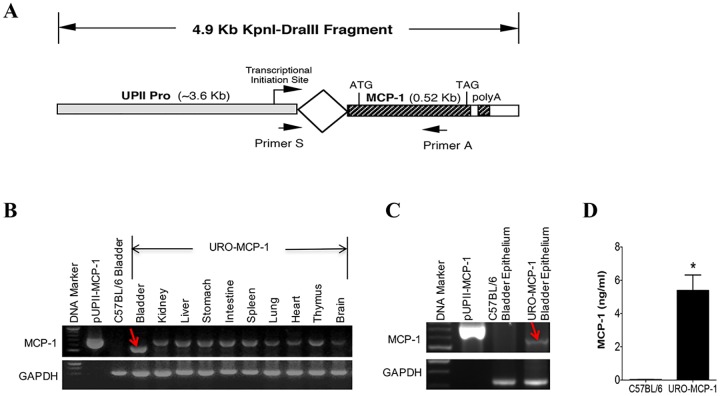
Transgenic MCP-1 gene and expression of MCP-1 in URO-MCP-1 mice. (A) Schematic illustration of the transgenic MCP-1 DNA construct. The mouse MCP-1 coding sequence with a secretory element (0.52Kb) was placed downstream to the UPII gene promoter (UPII Pro; ~3.6 Kb). An intron sequence was placed between the UPII Pro and the MCP-1 coding sequence. The targeting regions of the primer pair used for PCR genotyping and RT-PCR are indicated by arrows. ATG, a start codon; TAG, a stop codon; poly(A), an additional poly(A) site. (B) URO-MCP-1 mice express MCP-1 mRNA in the bladder. RT-PCR was performed on various tissues from an URO-MCP-1 mouse. MCP-1 mRNA product is indicated by a red arrow. GAPDH was used as an internal control. pUPII-MCP-1, a plasmid containing the transgenic MCP-1 DNA sequence (a control for MCP-1 genomic DNA). C57BL/6 bladder, a C57BL/6 mouse bladder (a negative control). (C) URO-MCP-1 mice express MCP-1 mRNA in the bladder epithelium. The bladder epithelium from an URO-MCP-1 mouse was processed for RT-PCR. MCP-1 mRNA product is indicated by a red arrow. GAPDH was used as an internal control. pUPII-MCP-1 served as a control for MCP-1 genomic DNA. C57BL/6 bladder epithelium served as a negative control. (D) URO-MCP-1 mice express MCP-1 protein in the urine. Urine was collected from both C57BL/6 (n = 5) and URO-MCP-1 mice (n = 12) and analyzed for MCP-1 by ELISA. Data are shown as mean ± s.d. *p<0.001 as compared to the urine of C57BL/6 mice.

The schematic structure of the transgenic MCP-1 construct is shown in Fig 1A. A plasmid containing the uroplakin II (UPII) gene promoter was kindly provided by Dr. T. Sun at the New York University School of Medicine [19]. The 3.6 Kb fragment containing the UPII gene promoter was excised and placed upstream to the mouse MCP-1 coding sequence (0.52 Kb). An intron sequence was inserted between the UPII gene promoter and the MCP-1 transgene to facilitate discriminating MCP-1 mRNA from its genomic DNA in RT-PCR analysis [20]. The 4.9 Kb Kpnl-DraIII DNA fragment consisting of the above-mentioned sequences and a poly A additional site was microinjected into fertilizing eggs (B6/SJL background; The Jackson Laboratory, Bar Harbor, Maine). Transgenic founders were backcrossed with C57BL/6 mice (Charles River Laboratories, Wilmington, MA) for 10 generations to generate C57BL/6 congenic URO-MCP-1 mice. Each generation was confirmed for the presence of the MCP-1 transgene by PCR genotyping using a sequence-specific primer pair (5’-CGAGGTCGACTGCAGAAG-3’ and 5’-TGAGGTGGTTGTGGAAAAGG-3’; 690 bp). PCR was performed for 40 cycles at 94°C for 30 seconds, 55°C for 30 seconds, and 72°C for one minute. The PCR products were analyzed by 1% agarose gel electrophoresis.

### Cystitis induction

Female mice (6–8 weeks old) were used due to a higher incidence of IC/BPS in females than males in humans and the easier feasibility of intravesical procedures in females. Mice were anesthetized by intraperitoneal (i.p.) injection with 100 μl of a mixture solution of ketamine (87.5 mg/kg) and xylazine (12.5 mg/kg). The bladder was then catheterized urethrally with a 24 gauge 3/4" long plastic intravenous catheter (Smiths Medical, Southington, CT), instilled with 1 μg of LPS (*E*. *coli* 055:B5, Sigma-Aldrich, St. Louis, MO) in 100 μl phosphate-buffered saline (PBS), and retained for 1 hour. Mice instilled with 100 μl PBS in the bladders served as controls.

### Bladder histology

Bladders were collected and processed for formalin fixation, paraffin embedment, section preparation, hematoxylin and eosin (H&E) staining, and photography as described previously [21]. Bladder inflammation was scored in a blinded manner based on infiltration of inflammatory cells in the lamina propria and the presence of interstitial edema as described previously: 1+ (mild infiltration with no or mild edema), 2+ (moderate infiltration with moderate edema), and 3+ (moderate to severe infiltration with severe edema) [21].

### Bladder immunohistochemistry

Bladder immunohistochemistry was performed as described previously [21]. Briefly, the bladders were fixed in 10% neutral formalin, embedded in paraffin, and cut into 5 μm sections. After antigen retrieval and blocking, slides were incubated with biotinylated rat anti-mouse F4/80 antibody (BioLegend, San Diego, CA; clone: CI:A3-1; rat IgG2b) or control biotinylated rat IgG2b (BioLegend, San Diego, CA; clone: RTK4530;) overnight. Slides were developed conventionally using streptavidin-horseradish peroxidase complex (SAv-HRP) and diaminobenzidine (DAB) substrate solution (BD PharMingen). After rinsing, slides were counterstained with hematoxylin solution and photographed using an Olympus BX-51 microscope.

### Reverse transcriptase-polymerase chain reaction (RT-PCR)

Total RNAs were extracted from the bladder and bladder epithelium using Qiagen RNeasy Mini Kit (Qiagen, Valencia, CA) as described previously [21]. cDNA was synthesized using Invitrogen SuperScript III Reverse Transcriptase (Invitrogen, Carlsbad, CA) and Oligo dT. PCR amplification was performed on cDNA products using Taq DNA polymerase (New England Biolabs, Ipswich, MA) and sequence-specific primer pairs for MCP-1 (5’-CGAGGTCGACTGCAGAAG-3’ and 5’-TGAGGTGGTTGTGGAAAAGG-3’; 560 bp), IL-1ß (5’-GCCCATCCTCTGTGACTCAT-3’ and 5’-AGGCCACAGGTATTTTGTCG-3’; 230 bp), IL-6 (5’-GTTCTCTGGGAAATCGTGGA-3’ and 5’-GGAAATTGGGGTAGGAAGGA-3’; 339 bp), tachykinin-1 (substance P precursor) (5’-GCCAATGCAGAACTACGAAA-3’ and 5’-GCTTGGACAGCTCCTTCATC-3’; 280 bp), nerve growth factor (NGF) (5’-CTGTGGACCCCAGACTGTTT-3’ and 5’-CACTGAGAACTCCCCCATGT-3’; 194 bp), and glyceraldehyde-3-phosphate dehydrogenase (GAPDH) (5’-GTTCCAGTATGACTCCACT-3’ and 5’-GTGCAGGATGCATTGCTG-3’; 321 bp). GAPDH was amplified for 25 cycles and other molecules were amplified for 40 cycles. The PCR products were run on a 2% agarose gel, stained with ethidium bromide, and imaged by Gel Doc EZ Imager (Bio-Rad Laboratories, Hercules, CA).

### Enzyme-linked immunosorbent assay (ELISA)

Urine was collected using micturition cages (see below) and levels of urinary MCP-1 were measured using ELISA with paired capture and detecting antibodies (R&D Systems, Minneapolis, MN).

### Voiding habit analysis

Mice were placed in individual micturition cages (Columbus Instruments, Columbus, OH) for 24-hour real time recording of voiding habits with 12-hour light and 12-hour dark cycles as described previously [22]. The cages consist of a fluid receptacle that funnels the captured urine droplets into a specimen freezer to prevent urine evaporation and chemical changes. A balance beneath the specimen freezer measures the weight of the collected urine. Mice had free access to drinking water but were restrained from solid food to prevent feces from interfering with measurement of urine output. The entire system was computer interfaced for automated data acquisition in 2-minute intervals using Oxymax software (Columbus Instruments). Urinary frequency, voided volume per micturition, and total urine volume were recorded.

### Pelvic pain analysis

Pelvic pain was assessed by quantifying referred tactile allodynia of the lower abdominal region in response to applied force with a series of calibrated von Frey filaments as described previously [22]. Mice were kept in individual Plexiglas chambers (6 x 10 x 12 cm) with a stainless steel wire grid floor and allowed to acclimate for 20 minutes before testing. Five individual filaments (Stoelting Co., Wood Dale, IL) with forces of 0.04, 0.16, 0.4, 1 and 4 grams were used in ascending order of force. The filament was applied perpendicularly to the skin for 1–2 seconds with intervals of 5 seconds between each stimulus for a total of 10 applications. Stimulation was confined to the lower abdominal area in the general vicinity of the bladder. A positive response to filament stimulation was considered when mice showed sharp abdominal retraction, instant licking or scratching of the stimulated area, or jumping. Response frequency was calculated as the percentage of positive response to each filament. Tactile sensitivity of the plantar region of the hind paw was assessed using the same calibrated von Frey filaments. The 50% withdrawal threshold was calculated and presented. A positive response to hind paw stimulation was defined as either a sharp withdrawal or licking of the tested paw.

### Statistical analysis

Results were analyzed using Statistics Package for Social Sciences (SPSS 13.0, Chicago, IL), and presented as mean ± s.d. for urinary MCP-1 levels and mean ± SEM for both voiding habit and pelvic pain changes. Data was compared using Student’s *t*-test (two groups) or ANOVA followed by LSD post hoc tests (multiple groups). A value of *p*<0.05 was considered statistically significant.

## Results

### URO-MCP-1 mice express MCP-1 in the bladder epithelium and urine

URO-MCP-1 mice were generated through microinjection of a 4.9 Kb DNA construct consisting of the UPII gene promoter fused to the mouse MCP-1 coding sequence with a secretory element (Fig 1A). The UPII gene promoter is an urothelium-specific promoter and facilitates the expression of MCP-1 by the bladder epithelium [19]. URO-MCP-1 mice were found to express MCP-1 mRNA in the bladder but not in other organs tested (Fig 1B). Further analysis indicated the expression of MCP-1 mRNA by the bladder epithelium of URO-MCP-1 mice but not that of wild-type C57BL/6 mice (Fig 1C). Urine from URO-MCP-1 mice contained a significantly higher level of MCP-1 protein (5.39 ± 0.93 ng/ml) compared to urine from wild-type C57BL/6 mice (0.022 ± 0.016 ng/ml) (Fig 1D). URO-MCP-1 mice appear healthy and do not spontaneously develop bladder inflammation and symptoms during their lifespan.

### URO-MCP-1 mice exhibit bladder hypersensitivity and develop bladder inflammation upon intravesical administration of a single sub-noxious dose of LPS

**Fig 2:**
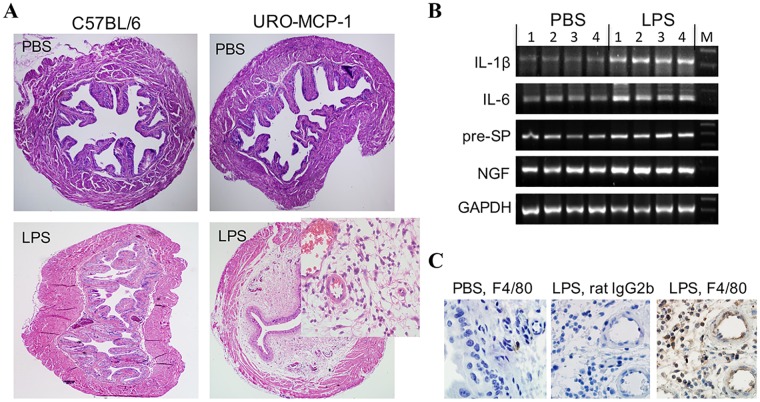
URO-MCP-1 mice exhibit bladder hypersensitivity and develop bladder inflammation upon intravesical administration of a single sub-noxious dose of LPS. (A) URO-MCP-1 mice develop bladder histopathology after cystitis induction. The bladders of both C57BL/6 (n = 7) and URO-MCP-1 mice (n = 8) were instilled with 100 μl PBS (*top panel*) or 1 μg of LPS in 100 μl PBS (*bottom panel*). After 24 hours the bladders were collected and processed for histological H&E staining. Magnification: x40 (the insert: x400). (B) The bladders of URO-MCP-1 mice express increased inflammatory factor mRNAs after cystitis induction. The bladder total RNAs were extracted 24 hours after intravesical PBS or LPS treatment and analyzed by RT-PCR for IL-1ß, IL-6, substance P precursor (pre-SP), and NGF mRNAs. GAPDH was used as an internal control. Four bladders for each group are presented. M: a 100 bp ladder. (C) The bladders of URO-MCP-1 mice show increased macrophage infiltration after cystitis induction. *Left panel*, the bladder of a mouse treated with intravesical PBS and stained with a rat anti-mouse F4/80 antibody (IgG2b); *Middle panel*, the bladder of a mouse treated with intravesical LPS and stained with a control rat IgG2b; *Right panel*, the bladder of a mouse treated with intravesical LPS and stained with a rat anti-mouse F4/80 antibody (IgG2b). The images are representative of 3–4 mice for each group. Magnification: x1,000.

The bladders of URO-MCP-1 mice show a hypersensitive response to intravesical LPS. Compared to wild-type C57BL/6 mice (n = 7; score: 0—+), URO-MCP-1 mice developed clear histological bladder inflammation (n = 8; score: ++—+++) 24 hours after intravesical instillation of a single sub-noxious dose of LPS (1 μg of LPS in 100 μl PBS) (Fig 2A, Table 1). Intravesical instillation of PBS (100 μl) did not induce bladder histological changes in either strain of mice. The LPS-treated bladders of URO-MCP-1 mice exhibited severe interstitial edema, mucosal hyperemia, and cellular infiltration in the lamina propria. In parallel with bladder histopathology, the inflamed bladders expressed elevated levels of mRNAs for IL-1ß, IL-6, substance P precursor (pre-SP), and NGF as detected by RT-PCR (Fig 2B). Immunohistochemistry revealed increased F4/80 positive cell (macrophages) infiltration in the inflamed bladders (Fig 2C). These observations indicate that the bladders of URO-MCP-1 mice are highly sensitive to minor irritants and develop exaggerated inflammation upon stimulation with a single sub-noxious dose of LPS. The bladder inflammation peaked at days 1–3 and diminished by day 7.

**Table 1:** Bladder response to a single sub-noxious dose of intravesical LPS.

	Bladder Histopathology			
	-	+	++	+++
C57BL/6 (n = 7)	6	1	0	0
URO-MCP-1 (n = 8)	0	0	3	5

### Bladder inflammation is associated with voiding dysfunction in URO-MCP-1 mice

**Fig 3:**
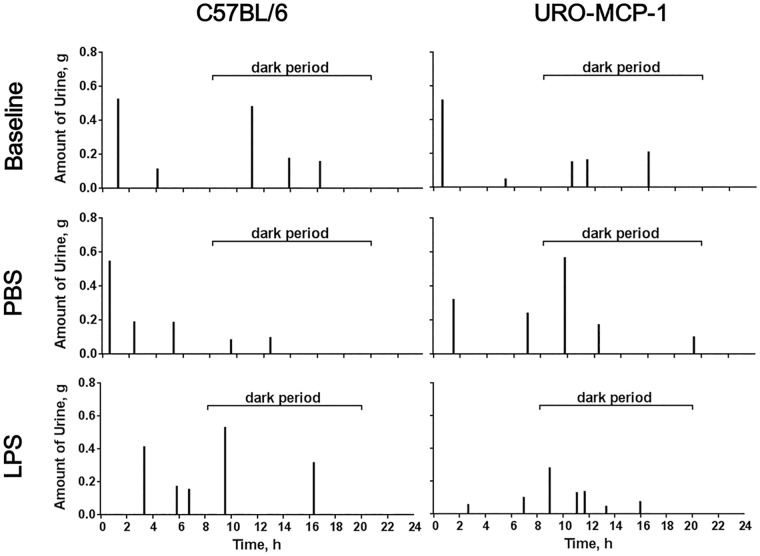
Bladder inflammation is associated with voiding dysfunction in URO-MCP-1 mice. Both wild-type C57BL/6 (*left panels*) and URO-MCP-1 mice (*right panels*) were treated intravesically with 100 μl PBS or 1 μg of LPS in 100 μl PBS and evaluated for voiding habits using micturition cages at 1, 3 and 7 days after intravesical treatment (see Table 2 and S2 Table). The baseline voiding habits were included for comparison (see S1 and S3 Tables). The results are representative of 5–7 mice for each of baseline, PBS-treated (day 1), and LPS-treated (day 1) groups in both mouse strains.

URO-MCP-1 mice were evaluated for voiding habits using micturition cages before (baseline) and 1, 3 and 7 days after intravesical PBS or LPS treatment (Table 2, S1 Table). For comparison, wild-type C57BL/6 mice were evaluated in parallel for voiding habits (S2 and S3 Tables). There were no significant changes in voiding habits after intravesical PBS treatment compared to baseline voiding habits for both URO-MCP-1 (S1 Table) and C57BL/6 mice (S3 Table). Our prior analysis also showed no significant differences in baseline voiding habits between C57BL/6 and URO-MCP-1 mice (S4 Table). While a single sub-noxious dose of LPS failed to induce clear changes in voiding habits in C57BL/6 mice (S2 Table), the LPS treatment induced significant changes in voiding habits in URO-MCP-1 mice at days 1 and 3 (Table 2). These changes included decreased average volume voided per micturition (day 1: 0.159±0.01 vs 0.303±0.03, *p* = 0.002; day 3: 0.21±0.01 vs 0.292±0.02, *p* = 0.01), decreased maximum volume voided per micturition (day 1: 0.325±0.03 vs 0.498±0.05, *p* = 0.026; day 3: 0.383±0.03 vs 0.57±0.07, *p* = 0.043), and increased total number of voids (day 1: 7.8±0.74 vs 4.143±0.40, *p* = 0.001; day 3: 7.833±0.60 vs 4.857±0.26, *p* = 0.003). The number of voids in the dark period was increased (day 1: 5.0±0.71 vs 2.143±0.46, *p* = 0.005; day 3: 5.0±0.37 vs 2.714±0.29, *p*<0.001). Fig 3 represents the typical voiding habits of C57BL/6 and URO-MCP-1 mice before (baseline) and one day after intravesical PBS or LPS treatment. The LPS-treated URO-MCP-1 mice remained voiding dysfunction at day 7 after cystitis induction, but changes did not reach statistical significance (Table 2). Total voided volumes in 24 hours were similar between PBS- and LPS-treated URO-MCP-1 mice (day 1: 1.201±0.106 vs 1.255±0.183, *p* = 0.791; day 3: 1.422±0.136 vs 1.657±0.167, *p* = 0.294; Day 7: 1.380±0.171 vs 1.299±0.080, *p* = 0.681). There were also no significant differences in the total voided volumes between PBS- and LPS-treated C57BL/6 mice (day 1: 1.474±0.157 vs 1.716±0.194, *p* = 0.36; day 3: 1.666±0.151 vs 1.561±0.218, *p* = 0.725; Day 7: 1.327±0.126 vs 1.294±0.135, *p* = 0.86). These observations indicate that URO-MCP-1 mice readily develop voiding dysfunction upon cystitis induction with a single sub-noxious dose of intravesical LPS.

**Table 2:** Changes in voiding habits after a single sub-noxious dose of intravesical LPS in URO-MCP-1 mice.

	Day 1		Day 3		Day 7	
	PBS (n = 7)	LPS (n = 5)	PBS (n = 7)	LPS (n = 6)	PBS (n = 6)	LPS (n = 7)
**Average volume voided per micturition, g**	0.303±0.03	0.159±0.01 (*p* = 0.002)	0.292±0.02	0.210±0.01 (*p* = 0.010)	0.259±0.02	0.205±0.02 (*p* = 0.053)
**Maximum volume voided per micturition, g**	0.498±0.05	0.325±0.03 (*p* = 0.026)	0.570±0.07	0.383±0.03 (*p* = 0.043)	0.467±0.06	0.336±0.03 (*p* = 0.056)
**Total number of voids**	4.143±0.40	7.800±0.74 (*p* = 0.001)	4.857±0.26	7.833±0.60 (*p* = 0.003)	5.333±0.49	6.429±0.30 (*p* = 0.075)
in light	2.000±0.31	2.800±0.49 (*p* = 0.176)	2.143±0.26	2.833±0.48 (*p* = 0.213)	2.000±0.26	2.429±0.20 (*p* = 0.212)
in dark	2.143±0.46	5.000±0.71 (*p* = 0.005)	2.714±0.29	5.000±0.37 (*p*<0.001)	3.333±0.42	4.000±0.31 (*p* = 0.220)

### Bladder inflammation is associated with pelvic pain in URO-MCP-1 mice

**Fig 4:**
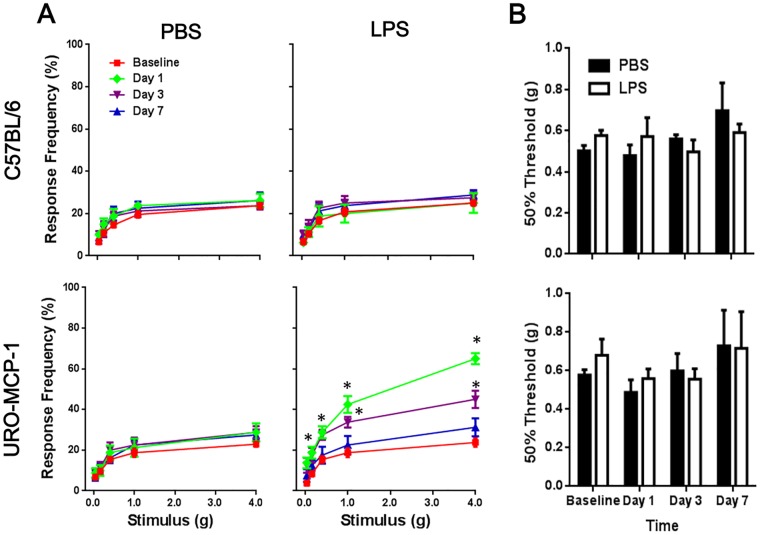
Bladder inflammation is associated with pelvic pain in URO-MCP-1 mice. (A) Both wild-type C57BL/6 (*top panels*) and URO-MCP-1 mice (*bottom panels*) were treated intravesically with 100 μl PBS or 1 μg of LPS in 100 μl PBS and evaluated for pelvic response to von Frey filament stimulation at 1, 3 and 7 days after intravesical treatment. The baseline pelvic responses were included for comparison. Data are shown as mean ± SEM percent of response frequency. *p<0.05 as compared to baselines. The results are representative of 8 mice in each group. (B) The same C57BL/6 and URO-MCP-1 mice exhibited no significant changes in tactile sensitivity (50% threshold) of the plantar region of the hind paw at 1, 3 and 7 days after intravesical PBS or LPS treatment as compared to baselines.

Both wild-type C57BL/6 and URO-MCP-1 mice were evaluated for pelvic pain using von Frey filament stimulation before (baseline) and 1, 3 and 7 days after intravesical PBS or LPS treatment (Fig 4A). There were no significant differences in baseline pelvic responses between C57BL/6 and URO-MCP-1 mice or in pelvic response changes after PBS treatment in both mouse strains. Intravesical LPS at a single sub-noxious dose induced no clear changes in pelvic response in C57BL/6 mice. However, the same LPS treatment induced significant changes in pelvic response in URO-MCP-1 mice at days 1 (0.16 g: 18.8±2.95 vs 10.0±2.67, *p* = 0.045; 0.4 g: 28.8±2.95 vs 18.8±3.5, *p* = 0.047; 1 g: 42.5±4.12 vs 21.2±4.41, *p* = 0.003; 4 g: 65.0±2.67 vs 28.8±4.41, *p*<0.001) and 3 (1 g: 33.8±2.63 vs 22.5±3.66, *p* = 0.026; 4 g: 45.0±4.23 vs 28.8±2.95, *p* = 0.007) as compared to baselines. There were no significant differences in tactile sensitivity (50% threshold) of the plantar region of the hind paw before (baseline) and 1, 3 and 7 days after intravesical PBS or LPS treatment in both wild-type C57BL/6 and URO-MCP-mice (Fig 4B), suggesting that the pain developed in intravesical LPS-treated URO-MCP-1 mice was restricted to the pelvis.

## Discussion

IC/BPS is one of the most refractory diseases in urology today and the effort to develop animal models that can reproduce the clinical correlates of the human disease is greatly needed for IC/BPS research. Since the etiology of IC/BPS remains elusive and many factors appear to be causative for the disease, animal models with diverse pathological pathways have been developed [22]. It is now generally agreed that a valid IC/BPS animal model must present, at minimum, pelvic or bladder nociception and/or voiding dysfunction such as urinary frequency [22]. In this study we created a novel transgenic cystitis model (URO-MCP-1) that secretes MCP-1 by the bladder epithelium and develops bladder inflammation upon intravesical instillation of a single sub-noxious dose of LPS. Besides bladder histopathology, the URO-MCP-1 model demonstrates both qualitative and quantitative changes in bladder functions such as increased pelvic pain sensitivity, increased urinary frequency, reduced average volume voided per micturition (urgency), and reduced maximum volume voided per micturition (bladder capacity). Because of the genetic stability of the incorporated transgene, the URO-MCP-1 model is stable and reproducible and provides a unique translational model for IC/BPS research.

URO-MCP-1 mice do not spontaneously develop bladder inflammation, pelvic pain and voiding dysfunction in the unmanipulated state, suggesting that the urothelial expression of MCP-1 alone is not sufficient to cause bladder inflammation and functional changes in these mice. However, URO-MCP-1 mice readily develop phenotypical and functional changes upon intravesical administration of a single sub-noxious dose of LPS. Our observation is similar to those observed in other MCP-1 transgenic models. Gunn and associates reported that transgenic mice expressing MCP-1 by type II alveolar epithelial cells showed no morphologic evidence of inflammation in the lung but exhibited enhanced inflammatory response upon treatment with either intraperitoneal LPS or intravenous yeast wall glucan [7]. Huang and associates reported that transgenic mice expressing MCP-1 by astrocytes only manifested neurological impairment and encephalopathy after treatment with intravenous pertussis toxin plus subcutaneous complete Freund’s adjuvant [8]. Similarly, Trujillo and associates reported that transgenic mice expressing MCP-1 by oligodendrocytes predisposed mice to a defective immune response to a minimally lethal neurotropic coronavirus and developed encephalitis following intracranial infection by the virus [9]. These observations indicated that development of inflammatory disorders is dependent on both genetic and environmental factors in the MCP-1 transgenic models.

Constitutive expression of MCP-1 by the bladder epithelium renders the bladders of URO-MCP-1 mice hypersensitive to otherwise sub-noxious irritative stimuli such as LPS. We have observed that URO-MCP-1 mice exhibit a much lower threshold trigger for producing exaggerated responses to a single sub-noxious dose of LPS as compared to wild-type C57BL/6 mice. Intravesical administration of LPS at 1 μg in 100 μl PBS efficiently induced profound bladder inflammation in URO-MCP-1 mice but not in wild-type C57BL/6 mice. This dose of LPS used for cystitis induction in URO-MCP-1 mice was only one-tenth of the dose commonly used for cystitis induction in wild-type C57BL/6 mice [23,24]. This feature of the URO-MCP-1 model is clinically relevant, as subclinical infection can be a causative factor for IC/BPS and patients with IC/BPS are often found to be hypersensitive to minor bladder irritants [25].

Inflammation plays a central role in the pathogenesis of IC/BPS and may directly affect bladder function in IC/BPS patients [25]. Our recent studies supported the fundamental role of inflammation in IC/BPS, as the production of Toll-like receptor 4 (TLR4) mediated proinflammatory cytokines IL-1ß and IL-6 was significantly associated with multiple IC/BPS pain indicators [26–28]. As a clinically relevant model, the URO-MCP-1 model develops bladder inflammation and functional changes such as pelvic pain, urinary frequency and urgency. In addition to bladder histopathology, the inflamed bladders expressed elevated mRNAs for proinflammatory cytokines IL-1ß and IL-6. Moreover, the inflamed bladders also expressed elevated mRNAs for NGF (a neurotrophic factor) and substance P precursor (a neurotransmitter), suggesting the presence of a strong neuro-immune interaction in the animal model. Multiple cell types including urothelial cells, macrophages, mast cells, and neurons are known to express these inflammatory factors, reflecting an ongoing local inflammatory response in the bladders of URO-MCP-1 mice. All these inflammatory factors have been detected in the urine and/or bladder tissues of IC/BPS patients [29].

The URO-MCP-1 model described in this study represents an acute cystitis model. Future studies will focus on extending this model to a chronic cystitis model to more closely mimic human IC/BPS. The other limitation of the present study is the lack of direct evaluation of bladder nociception. Although our data indicate the presence of increased pelvic pain sensitivity, we will continue demonstrating bladder pain by using more specific methods such as the bladder distention-evoked visceromotor response (VMR) method [22]. Future studies are also warranted to investigate the role of TLR4 in the URO-MCP-1 model and use this model for therapeutic development.

## Conclusions

The URO-MCP-1 model demonstrates bladder hypersensitivity, pelvic pain, and voiding dysfunction, providing a novel model for IC/BPS research.

## Supporting Information

S1 Table Voiding habits in URO-MCP-1 mice—baseline versus intravesical PBS treatment. (DOCX)

S2 Table C57BL/6 mice exhibit no significant changes in voiding habits after a single sub-noxious dose of intravesical LPS. (DOCX)

S3 Table Voiding habits in C57BL/6 mice—baseline versus intravesical PBS treatment. (DOCX)

S4 Table Baseline voiding habits—comparison between C57BL/6 and URO-MCP-1 mice. (DOCX)
