# Supplementary material for: Transgenic Mice Expressing MCP-1 by the Urothelium Demonstrate Bladder Hypersensitivity, Pelvic Pain and Voiding Dysfunction: A Multidisciplinary Approach to the Study of Chronic Pelvic Pain Research Network Animal Model Study
Source: PLoS One. 2016 Sep 29;11(9):e0163829. doi: 10.1371/journal.pone.0163829 (PMC5042429; doi:10.1371/journal.pone.0163829)
Supplement: S1 Table — (DOCX) [file pone.0163829.s001.docx]

**S1 Table. Voiding habits in URO-MCP-1 mice – baseline versus intravesical PBS treatment**

|  | Baseline  (n=7) | PBS*  (n=7) | *p*-value |
| --- | --- | --- | --- |
| Average volume voided per micturition, g | 0.281 ± 0.0245 | 0.303 ± 0.030 | 0.579 |
| Maximum volume voided per micturition, g | 0.493 ± 0.043 | 0.498 ± 0.050 | 0.943 |
| Total number of voids | 5.000 ± 0.436 | 4.143 ± 0.404 | 0.175 |
| in light | 1.857 ± 0.261 | 2.000 ± 0.309 | 0.730 |
| in dark | 3.286 ± 0.286 | 2.143 ± 0.459 | 0.056 |
| Total volume of voids, g | 1.371 ± 0.134 | 1.201 ± 0.106 | 0.340 |

***** 24 hours after intravesical PBS treatment
