# Supplementary material for: Transgenic Mice Expressing MCP-1 by the Urothelium Demonstrate Bladder Hypersensitivity, Pelvic Pain and Voiding Dysfunction: A Multidisciplinary Approach to the Study of Chronic Pelvic Pain Research Network Animal Model Study
Source: PLoS One. 2016 Sep 29;11(9):e0163829. doi: 10.1371/journal.pone.0163829 (PMC5042429; doi:10.1371/journal.pone.0163829)
Supplement: S2 Table — (DOCX) [file pone.0163829.s002.docx]

**S2 Table.** **C57BL/6 mice exhibit no significant changes in voiding habits after a single sub-noxious dose of intravesical LPS**

|  |  | **Day 1** | |  | **Day 3** | |  | **Day 7** | |
| --- | --- | --- | --- | --- | --- | --- | --- | --- | --- |
|  |  | **PBS** (n=5) | **LPS** (n=5) |  | **PBS** (n=5) | **LPS** (n=7) |  | **PBS** (n=8) | **LPS** (n=8) |
| **Average volume voided per micturition, g** |  | 0.280 ± 0.036 | 0.286 ± 0.027  (*p*=0.904) |  | 0.291 ± 0.024 | 0.284 ± 0.032  (*p*=0.868) |  | 0.271 ± 0.013 | 0.272 ± 0.034  (*p*=0.968) |
| **Maximum volume voided per micturition, g** |  | 0.589 ± 0.103 | 0.524 ± 0.064  (*p*=0.608) |  | 0.540 ± 0.037 | 0.537 ± 0.051  (*p*=0.967) |  | 0.442 ± 0.031 | 0.431 ± 0.039  (*p*=0.822) |
| **Total number of voids** |  | 5.400 ± 0.510 | 6.000 ± 0.316  (*p*=0.347) |  | 5.600 ± 0.678 | 5.429 ± 0.202  (*p*=0.819) |  | 4.875 ± 0.350 | 5.000 ± 0.423  (*p*=0.823) |
| in light |  | 1.800 ± 0.374 | 2.600 ± 0.51  (*p*=0.242) |  | 1.600 ± 0.245 | 2.143 ± 0.143  (*p*=0.187) |  | 1.625 ± 0.263 | 1.875 ± 0.398  (*p*=0.609) |
| in dark |  | 3.600 ± 0.678 | 3.400 ± 0.510  (*p*=0.82) |  | 4.000 ± 0.548 | 3.286 ± 0.184  (*p*=0.099) |  | 3.250 ± 0.250 | 3.125 ± 0.398  (*p*=0.794) |
