# Supplementary material for: Transgenic Mice Expressing MCP-1 by the Urothelium Demonstrate Bladder Hypersensitivity, Pelvic Pain and Voiding Dysfunction: A Multidisciplinary Approach to the Study of Chronic Pelvic Pain Research Network Animal Model Study
Source: PLoS One. 2016 Sep 29;11(9):e0163829. doi: 10.1371/journal.pone.0163829 (PMC5042429; doi:10.1371/journal.pone.0163829)
Supplement: S3 Table — (DOCX) [file pone.0163829.s003.docx]

**S3 Table. Voiding habits in C57BL/6 mice – baseline versus intravesical PBS treatment**

|  | Baseline  (n=5) | PBS*  (n=5) | *p*-value |
| --- | --- | --- | --- |
| Average volume voided per micturition, g | 0.324 ± 0.030 | 0.280 ± 0.036 | 0.369 |
| Maximum volume voided per micturition, g | 0.515 ± 0.050 | 0.589 ± 0.103 | 0.490 |
| Total number of voids | 5.143 ± 0.261 | 5.400 ± 0.510 | 0.635 |
| in light | 1.571 ± 0.202 | 1.800 ± 0.374 | 0.574 |
| in dark | 3.571 ± 0.202 | 3.600 ± 0.678 | 0.969 |
| Total volume of voids, g | 1.669 ± 0.183 | 1.474 ± 0.157 | 0.462 |

***** 24 hours after intravesical PBS treatment
