# Supplementary material for: Transgenic Mice Expressing MCP-1 by the Urothelium Demonstrate Bladder Hypersensitivity, Pelvic Pain and Voiding Dysfunction: A Multidisciplinary Approach to the Study of Chronic Pelvic Pain Research Network Animal Model Study
Source: PLoS One. 2016 Sep 29;11(9):e0163829. doi: 10.1371/journal.pone.0163829 (PMC5042429; doi:10.1371/journal.pone.0163829)
Supplement: S4 Table — (DOCX) [file pone.0163829.s004.docx]

**S4 Table. Baseline voiding habits – comparison between C57BL/6 and URO-MCP-1 mice**

|  | C57BL/6  (n=40) | URO-MCP-1 (n=43) | *p*-value |
| --- | --- | --- | --- |
| Average volume voided per micturition, g | 0.288 ± 0.011 | 0.281 ± 0.012 | 0.703 |
| Maximum volume voided per micturition, g | 0.506 ± 0.023 | 0.526 ± 0.023 | 0.540 |
| Total number of voids | 4.875 ± 0.153 | 5.047 ± 0.173 | 0.462 |
| in light | 1.550 ± 0.107 | 1.628 ± 0.100 | 0.596 |
| in dark | 3.325 ± 0.170 | 3.442 ± 0.161 | 0.618 |
| Total volume of voids, g | 1.406 ± 0.077 | 1.356 ± 0.048 | 0.586 |
